# *SORT1* Mutation Resulting in Sortilin Deficiency and p75^NTR^ Upregulation in a Family With Essential Tremor

**DOI:** 10.1177/1759091415598290

**Published:** 2015-08-20

**Authors:** Elena Sánchez, Alberto Bergareche, Catharine E. Krebs, Ana Gorostidi, Vladimir Makarov, Javier Ruiz-Martinez, Alejo Chorny, Adolfo Lopez de Munain, Jose Felix Marti-Masso, Coro Paisán-Ruiz

**Affiliations:** 1 Department of Neurology, Icahn School of Medicine at Mount Sinai, New York, NY, USA; 2 Biodonostia Research Institute, University of the Basque Country, San Sebastián, Gipuzkoa, Spain; 3 Department of Neurology, Hospital Universitario Donostia, San Sebastián, Guipuzcoa, Spain; 4 Centro de investigación biomédica en Red para enfermedades Neurodegenerativas (CIBERNED), Madrid, Spain; 5 Memorial Sloan-Kettering Cancer Center, New York, NY, USA; 6 Department of Medicine, The Immunology Institute, Icahn School of Medicine at Mount Sinai, New York, NY, USA; 7 Department of Neurosciences, University of the Basque Country, San Sebastián, Guipuzcoa, Spain; 8 Departments of Psychiatry and Genetics and Genomic Sciences, Icahn School of Medicine at Mount Sinai, New York, NY, USA; 9 Friedman Brain and Mindich Child Health and Development Institutes, Icahn School of Medicine at Mount Sinai, New York, NY, USA

**Keywords:** *SORT1* mutation, tremor, sortilin downregulation, p75^NTR^ upregulation

## Abstract

*These authors contributed equally to this work.Essential tremor (ET) is the most prevalent movement disorder affecting millions of people in the United States. Although a positive family history is one of the most important risk factors for ET, the genetic causes of ET remain unknown. In this study, whole exome sequencing and subsequent approaches were performed in a family with an autosomal dominant form of early-onset ET. Functional analyses including mutagenesis, cell culture, gene expression, enzyme-linked immunosorbent, and apoptosis assays were also performed. A disease-segregating mutation (p.Gly171Ala), absent in normal population, was identified in the *SORT1* gene. The p.Gly171Ala mutation was shown not only to impair the expression of its encoding protein sortilin but also the mRNA levels of its binding partner p75 neurotrophin receptor that is known to be implicated in brain injury, neuronal apoptosis, and neurotransmission.

*These authors contributed equally to this work.

## Introduction

Essential tremor (ET) is one of the most common neurological disorders among adults whose incidence increases with age. Albeit its core motor symptom is an 8- to 12-Hz kinetic tremor of the arms, some patients with ET also develop other motor and nonmotor manifestations, including parkinsonism, myoclonus, dystonia, cerebellar dysfunction, sensory abnormalities, sleep disorders, and cognitive and psychiatric features (Louis, 2010). Despite many magnetic resonance imaging (MRI) studies have supported the hypothesis that the abnormalities of the cerebellothalamo-cortical motor pathway and the fronto-parietal circuit are involved in the functional pathological changes of ET (Bagepally et al., 2012), there is still a controversy as to whether there is an underlying neurodegenerative process of the cerebellum in ET (Buijink et al., 2013; Symanski et al., 2014). Consequently, there is growing evidence that ET may not be a single disease but rather a family of diseases (Benito-Leon, 2014).

Although familial aggregation has been long reported in ET, with 50% to 70% of patients having a familial form of ET, and a positive family history being one of the most important risk factor for ET (Louis et al., 2001), the genetic basis of ET remain elusive. This is partly due to misdiagnosis is a common feature in ET, with 37% to 50% of ET patients reported to be misdiagnosed (Schrag et al., 2000). Genetic variants within *LINGO1* (MIM *#*609791), *DRD3* (MIM *#*126451), and *HS1-BP3* (MIM *#*609359) genes have been reported to have susceptibility for developing ET; however, these associations have not been consistently replicated (Zimprich, 2011; Kuhlenbaumer et al., 2014). Through genome-wide association and whole exome sequencing (WES) analyses, *SLC1A2* (MIM #600300) and *FUS* (MIM #137070) genes have been reported to respectively carry susceptibility or causative alleles for ET (Merner et al., 2012; Thier et al., 2012). While the disease-associated mutation identified in the *FUS* gene was only present in 54% of individuals classified as “possible” ET, it fully segregated with disease in individuals with definite and probable ET (Merner et al., 2012). More recently, a disease-segregating mutation (p.G399S) in the *HTRA2* gene has been reported in a large kindred featuring both ET and Parkinson’s disease (Unal Gulsuner et al., 2014). Taken together, these clinical barriers associated with ET make traditional gene discovery approaches not succeed in the identification of the causal gene defect. Therefore, in an attempt to identify a causative gene for ET, we performed WES and subsequent functional analyses in a family featuring an autosomal dominant form of early-onset ET.

## Materials and Methods

### Subjects

**Figure 1.**
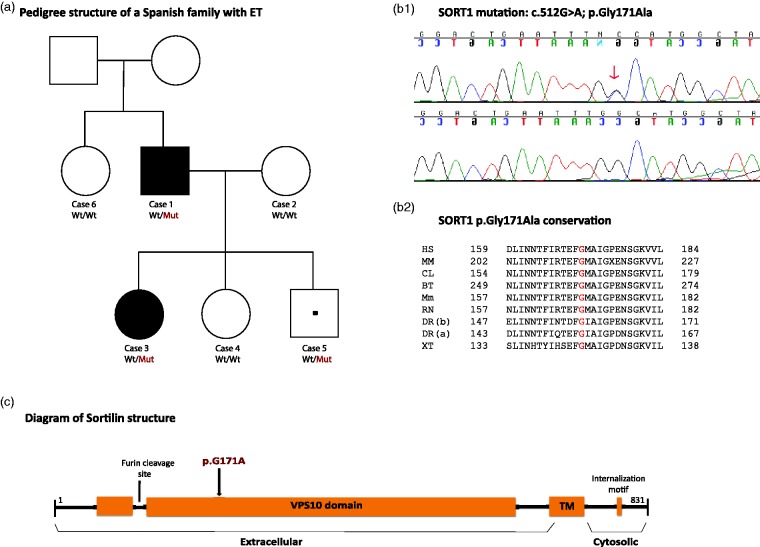
Pedigree structure of a Spanish family with ET identified with a pathogenic SORT1 mutation. (a): Definitive affected cases are represented with black filled square (male) and circle (female) and a dot inside a square represents an individual male at risk. Wt/Mut indicates heterozygous carrier for the mutant allele while Wt/Wt indicates noncarrier. (b1): Sanger chromatograms of the human reference sequence (bottom) and heterozygous mutant sequence (top) with a red arrow highlighting the pathogenic mutation. (b2): The conservation of the p.G171A mutation among other orthologous is shown. HS: H. Sapiens; MM: M. Mulatta; CL: C. Lupus; BT: B. Taurus; Mm: M. musculus; RN: R. Norvegicus; DR: D. Rerio; XT: X. Tropicalis. 1(c): Diagram of sortilin structure. All protein domains are shown. TM: Transmembrane domain.

A Spanish ET family consisting of an affected father, an unaffected paternal aunt, a healthy mother as well as one affected and two unaffected siblings was clinically examined and subject to WES approaches (Figure 1(a)). Subjects were recruited from a descriptive study of familiar and sporadic ET cases and controls carried out in the Movement Disorders Unit at the Donostia University Hospital (San Sebastian, Spain). Genomic DNA samples from all family members in addition to samples from another 28 familial and 62 sporadic ET cases (*n* = 90) were available for study. A cohort composed of 188 control chromosomes of Spanish individuals without family history of any movement disorders was also available. The age at sample collection of the control cohort ranged from 60 to 93 years with an average of 69.1 years. The local ethics committee at the Donostia University Hospital approved this study and informed consent was obtained from all participants. DNA samples from all participating members were isolated from whole blood using standard procedures. All methods were carried out in accordance with the approved guidelines.

### Clinical Examination

All participants in this study underwent a series of structured questionnaires and a comprehensive neurological and neuropsychological assessment undertaken at the Movement Disorders Unit by three experienced movement disorder specialist (JFM-M, JRM, and AB). The following standardized protocol was used: (a) demographic variables; (b) personal and family history and general medical health (Cumulative Illness Rating Scale score [range: 0–42], total number of prescription medications); (c) neurological assessment: a subjective motor complains questionnaire, the Activities-specific Balance Confidence scale, a motor examination including UPDRS part III and a general examination in order to detect dystonia, myoclonus, ataxia, and polyneuropathy as well as a specific tremor exploration that includes one test for postural tremor and five for kinetic tremor; (d) the use of medication (yes vs. no); (e) additional variables of interest (e.g., age of symptom onset); (f) the SCOPA-AUT assessment of autonomic dysfunction (Visser et al., 2004); and (g) the Pittsburgh sleep quality index (Buysse et al., 1989). The Montreal Cognitive Assessment (MoCA) that assesses different cognitive domains (http://www.mocatest.org/) was used to determine possible cognitive dysfunction and DSM-IV criteria were used for diagnosis of depression and anxiety disorders (American Psychiatric Association, 1994). In addition to the standard clinical exploration described above, evaluation of ET was carried out by recording the drawing of an “Archimedes spiral” (Elble et al., 1990) and using the Fahn–Tolosa–Marin tremor rating scale (TRS; Fahn et al 2003). Surface electromyography (EMG) was recorded from wrist extensor and flexor muscles using surface electrodes placed over the muscle bellies 3 cm apart. The filters were set with a bandpass of 10 Hz to 1 kHz. A triaxial accelerometer was placed over the first dorsal interossei muscle of the hand. In an effort to minimize diagnostic pitfalls, clinical criteria were comprehensively reviewed by the Consensus Statement of the Movement Disorder Society on Tremor (Deuschl et al., 1998) and the Washington Heights–Inwood Genetic Study of Essential Tremor criteria (Louis et al., 1997; Benito-Leon and Louis, 2006). Each patient received a diagnosis of ET from a movement disorders neurologist after the first evaluation which subsequently was confirmed by consensus with the rest of the team based on the review of the clinical data and electrophysiological records from the second evaluation using the formerly described diagnostic criteria. Criteria for definitive ET included abnormal bilateral postural or kinetic tremor of the hands in the absence of other neurological symptoms.

### WES Approaches

Four DNA samples from two affected (Cases 1 and 3) and two unaffected cases (Cases 2 and 4) were subject to WES analyses (Figure 1(a)). The SureSelect Human All exon 50 Mb exon-capture kit was used for library enrichment (Agilent Technologies Inc., Santa Clara, CA) and captured libraries were sequenced on the HiSeq2000 according to the manufacturer’s instructions for paired-end 100-bp reads (Illumina Inc, San Diego, CA), using a single flow cell lane per sample. Sequencing data were put through a computational pipeline for WES data processing and analysis following the general workflow adopted by the 1000 genomes project (DePristo et al., 2011) and as described elsewhere (Marti-Masso et al., 2013). Each exome’s statistics were conducted in PICARD (http://picard.sourceforge.net/).

### Filtering of Common Genetic Variation

Any potential mutation observed as common variation (frequency > 5%) in the latest dbSNP137 build, 1000 Genomes Project Phase 1, other public databases, such as the Exome Variant Server of the National Heart, Lung, and Blood Institute Exome Sequencing Project (http://evs.gs.washington.edu/EVS/; Exome Variant Server, 2015), or exomes generated in house (Marti-Masso et al., 2013) was removed from further analyses. Only genetic variants mapping to coding and splice site regions were considered causative.

### Prediction of Mutation Pathogenicity

The pathogenicity of each novel disease-segregating mutation was predicted by four computational methods: MutPred (http://mutpred.mutdb.org/), SNPs&Go (http://snps-and-go.biocomp.unibo.it/snps-and-go/), Mutation taster (http://www.mutationtaster.org/), and SIFT (http://sift.jcvi.org/). The NCBI HomoloGene database was used to examine the conservation of novel SNVs identified in different species (http://www.ncbi.nlm.nih.gov/homologene). The professional Human Gene Mutation Database (https://portal.biobase-international.com/hgmd/pro/start.php) and the NCBI ClinVar database (http://www.ncbi.nlm.nih.gov/clinvar/) were used to determine if novel single nucleotide variations (SNVs) were already known to be associated with disease-related phenotypes.

### Gene Screening Analyses

Genomic primers for Polymerase Chain Reaction (PCR) amplifications of the entire coding region and intron–exon boundaries of *SORT1*, *LAPTM5* exon 5, and *GRIN2D* exon 13 were designed using a primer design public website (http://ihg.gsf.de/ihg/ExonPrimer.html; primer sequences available upon request). All purified PCR products were sequenced in both forward and reverse directions by Sanger sequencing using Applied Biosystems BigDye terminator v3.1 sequencing chemistry as per the manufacturer’s instructions and analyzed as described elsewhere (Marti-Masso et al., 2013).

### Mutagenesis and Cell-Culture Assays

Human embryonic kidney cells HEK293 were cultured in Dulbecco’s modified eagle medium supplemented with 10% fetal bovine serum and 1% penicillin/streptomycin. The *SORT1* p.Gly171Ala (p.G171A) mutation was introduced into the human pCMV6-XL5-*SORT1* wild-type plasmid (Origene, Rockville, MD) by site-directed mutagenesis (QuikChangeII, Agilent Technologies, Santa Clara, CA). All generated constructs were verified in both directions by Sanger sequencing and using the human sequence GRCh37.p13 as a reference.

HEK293 cells were grown in 96-well and 12-well tissue culture plates. After reaching confluency (∼80%), cells were transiently transfected using FuGene 6 Transfection Reagent (Promega, Madison, WI) with 0.1 µg of either pCMV6-XL5-*SORT1* wild-type or mutant human plasmids. After 24 hr, culture media was replaced and cells were treated independently with 10 ng/ml or 30 ng/ml of two different pro-neurotrophics factors: pro-nerve growth factor (proNGF) and pro-brain-derived neurotrophic factor (proBDNF), or with a combination of both of them (Novoprotein, Summit, NJ). Cells were then incubated at 37℃ for 72 hr except for apoptosis assays in which a 16 h incubation was used. Conditioned media was collected and cells were harvested.

### Gene Expression Analyses

RNA extraction from HEK293 cells was performed using the RNeasy Plus Mini Kit (Qiagen, Hilden, Germany) as per manufacturer’s instructions. Extracted RNA was transcribed into cDNA with SuperScript II reverse transcriptase (Life Technologies, Grand Island, NY). Gene expression was carried out by qPCR using an Eco Real-Time PCR System (Illumina, San Diego, CA), SYBRGreen PCR master mix (Applied Biosystems, Foster City, CA), and the following oligonucleotide sequences: SORT1-F 5′-GGGGACACATGGAGCATGG-3′ and SORT1-R 5′-GGAATAGACAATGCCTCGATCAT-3′ for SORT1 expression, B2MF1-F 5′-GGCCGAGATGTCTCGCTCCG-3′ and B2MR1 5′-TTGGAGTACGCTGGATAGCCTCC-3′ for B2M, and p75-F 5′-CCTACGGCTACTACCAGGATG-3′ and p75-R 5′-CACACGGTGTTCTGCTTGT-3′ for p75^NTR^. Samples were run in triplicates, analyzed using the standard curve method, and normalized to *B2M* housekeeping gene using the 2^−ΔΔ^*^C^*^T^ method (Livak and Schmittgen, 2001).

### Immunoblot Analyses

Lysates from cells were prepared in radioactive immunoprecipitation assay buffer and treated with protease inhibitor cocktail. Equal amounts of proteins were added to Laemmli’s buffer (Bio-Rad, Hercules, CA), heated for 5 min at 95℃, and loaded into a 15-wells 4% to 12% Bis-Tris gel (Invitrogen, Carlsbad, CA). Proteins were then transferred to PVDF membranes (Bio-Rad, Hercules, CA), blocked with 5% nonfat dry milk in Phosphate Buffered Saline with Tween-20 (PBST), and incubated with primary antibody anti-SORT1 (1 µg/ml; (Abcam, Cambridge, MA; ab16640) and secondary antibody anti-Rabbit conjugated to horseradish peroxidase (1:5,000; Abcam; ab6721). Anti-GAPDH antibody (1:5000, Trevigen, Gaithersburg, MD) was used as loading control. Protein expression was detected by using Lumminata Forte (Millipore, Billerica, MA), gel images were obtained using a G:BOX Chemi image analyzer (Syngene, Frederick, MD), and densitometric analyses were performed through the ImageJ software (imagej.nih.gov).

### Enzyme-Linked Immunosorbent Assay (ELISA)

Human progranulin (hPGRN) in conditioned medium from wild-type and mutant HEK-293 cells was detected using the DuoSet ELISA Development System kit (R&D Systems, Minneapolis, MN) following the manufacturer’s protocol. The hPGRN from five human samples (Cases 2–6; Figure 1(a)) was measured using the hPGRN ELISA kit from Adipogen (San Diego, CA). Absorbance values were measured using a M5 multimodal plate reader (Molecular Devices, Sunnyvale, CA) at wavelengths of 450 nm and 560 nm.

### Apoptosis Assay by Flow Cytometry Analysis

Quantitative analysis of apoptotic cell death was done using the Alexa Fluor® 488 Annexin V/Dead Cell Apoptosis Kit according to the manufacturer’s protocol (Invitrogen, Carlsbad, CA). HEK293 cells were treated with 30 ng/ml of proneurotrophins (proNTs) for 16 hr; cells were then harvested, stained with Alexa Fluor® 488-Annexin V and PI in annexin-binding buffer, and analyzed by flow cytometry using a BD LSR II flow cytometer and the BD FACSDiva software (BD Bioscience, San Jose, CA). Cells stained with only Annexin V were considered as being in early apoptosis, while cells stained with both Annexin V and PI were considered to be in late apoptosis or necrotic stage. HEK293 cells treated with 2 mM of hydrogen peroxide were used as positive control for apoptosis. Data analysis was performed using the FlowJo software version 9.3.2 (TreeStar, Ashland, OR).

### Statistical Analyses

Statistical analyses were performed using the GraphPad Prism software version 6.00 (GraphPad, La Jolla, CA). Data on graphs are presented as mean ± *SEM*. Statistical differences between wild-type and mutant were calculated by using the Mann-Whitney nonparametric *U* test. For *SORT1* mRNA expression using different proNT concentrations, statistical significance was determined using a linear regression model. Values of *p* ≤ .0001 were considered highly significant (****).

## Results

### Phenotypic Examination of a Spanish Family With ET

The reported ET family, who consisted of two “definitive” affected individuals as well as four unaffected members (Figure 1(a)), featured postural tremor of both hands with slow progression since childhood. Psychiatric features, such as depression and anxiety, were found in one unaffected member. Detailed clinical descriptions of all family members examined are described below.

#### Case 1

**Figure 2.**
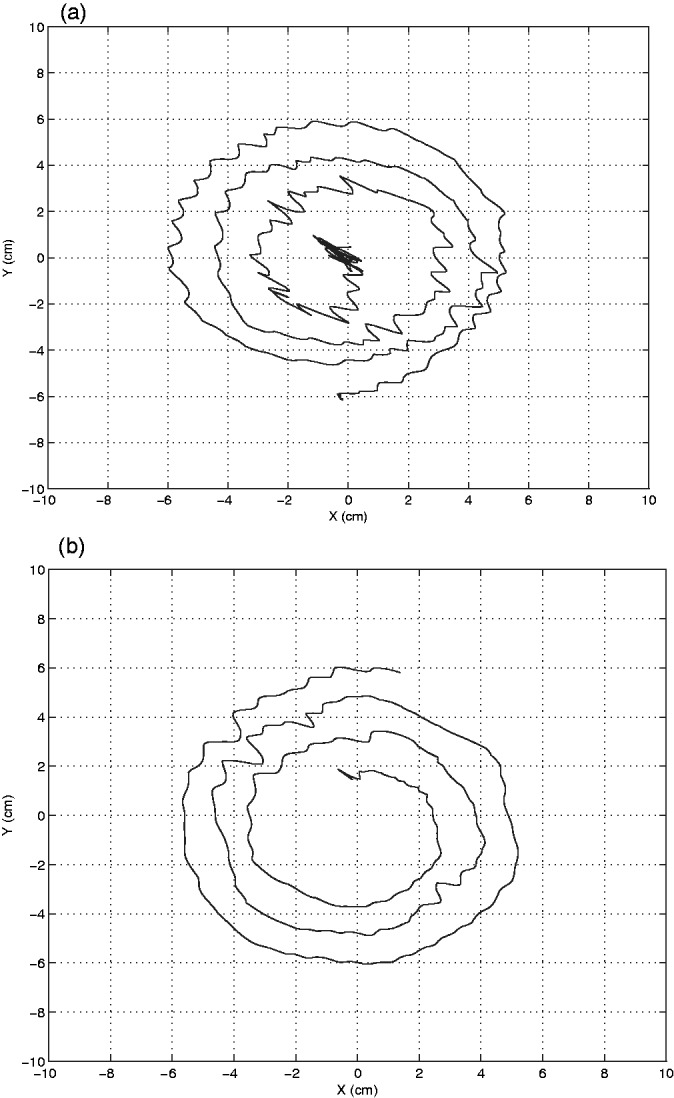
Archimedes spirals. Right hand spirals of affected Case 1 (a) and Case 3 (b) are shown. Archimedes spirals were drawn on a digitizing tablet.

This is an affected male who was first seen in 2006 with postural and action tremor of both hands and without voice, head, or rest tremors. He reported to have tremor with slow progression since childhood. No other signs of motor impairment suggestive of parkinsonism, myoclonus, dystonia, ataxia, and polyneuropathy were identified, and he did not show any sign of autonomic involvement, sleep disorder (insomnia, drowsiness, REM sleep behavior disorder (RBD)), anxiety, or depression. He did not take any medication for tremor, but his tremor improved with alcohol intake. He reported hypercholesterolemia and had a myocardial infarction in 2000 and an atherothrombotic left pontine infarction in 2001. In 2008, he died of head injury with a left subdural hematoma and intracranial fronto-temporal hemorrhage at 73 years of age. He scored 36 in the TRS scale and had a MoCA value of 27/30 (normal value > 25). His drawing of right hand spiral is shown in Figure 2(a).

#### Case 2

This is the wife of case 1 who did not show any sign of tremor or other neurological symptoms.

#### Case 3

This is a 48-year-old woman who suffers from tremor of both hands since childhood. During examination, she showed symmetric postural tremor involving hands without resting or action tremors and without affecting head, voice, and trunk. Her gait was normal and no clinical signs of parkinsonism, myoclonus, dystonia, ataxia, and polyneuropathy were found. She did not present any sign of autonomic involvement, sleep disorder (insomnia, drowsiness, RBD), anxiety, or depression. She scored 11 in the TRS scale and showed normal cognitive status with a MoCA value of 30/30 and a normal brain MRI. EMG recorded a postural tremor of 9.5 Hz of frequency and 46 mV of amplitude. Her total cholesterol was 244 mg/dl and low-density lipoprotein (LDL) cholesterol levels were slightly elevated (176 mg/dl). Her drawing of right hand spiral is shown in Figure 2(b).

#### Case 4

This is a 46-year-old woman who reported having mild tremor of both hands during her childhood when she was a regular tobacco smoker, but it greatly improved after smoking withdrawal. On a recent examination, she showed no tremor and a normal cognitive status (MoCA = 30/30). Her brain MRI, total cholesterol, and LDL levels were normal.

#### Case 5

This is a 43-year-old man who apparently has not reported tremor or an EMG-recorded tremor. His brain MRI was normal and showed a MoCA value of 30/30. He has high levels of total cholesterol and LDL and is under treatment with atorvastatine 20 mg/day.

#### Case 6

This is 67-year-old woman who has had anxiety and depression symptoms for 15 years and is being treated with 20 mg/day of paroxetine. On examination, she showed no tremor and her cognitive assessments (MoCA = 30/30) and LDL levels were normal.

None of the unaffected cases (Cases 4, 5, 6) showed any sign of motor involvement suggestive of parkinsonism, myoclonus, dystonia, ataxia, and polyneuropathy, nor nonmotor symptoms such as autonomic involvement, sleep disorders, anxiety, or depression.

### WES and Subsequent Analyses

WES approaches performed in four family members captured between 91.33% (Case 1) and 88.30% (Case 2) of the target exome at 20-fold coverage or higher. Approximately 1,000 novel, coding SNVs, including only missense and nonsense genetic variants, were identified in each family member sequenced: 1062 SNVs were identified for Case 3, 1,024 for Case 1, 1,006 for Case 2, and 884 for Case 4. After adequate filtering and by keeping SNVs only shared by both affected individuals, we were left with six SNVs as potential disease-associated mutations. Two of these were not validated through Sanger sequencing and the other four were also present in the youngest family member (Case 5), who is apparently unaffected at his 43 years of age. Because the existence of a variable age at onset is common in families with tremor, every unaffected individual younger than 50 years should still be considered at risk (Merner et al., 2012). All four novel SNVs were then tested in 188 ethnicity-matched normal chromosomes, but only one was found present in the control population. A DNA sample from an additional unaffected member (Case 6) was then acquired and tested for these three novel SNVs; she was found to be a noncarrier for any of them. By using four different computational programs, only two SNVs were predicted to be deleterious by all programs. Although one of this (p.Gly171Ala) was reported with very low frequency (5.769E-05) in the Exome Aggregation Consortium (ExAC) that contains exome data for a variety of rare diseases including rare neuromuscular diseases (ExAC, 2015), none of them were reported in the European population. These two SNVs were also highly conserved across different species while the other one reported in European population (ExAC data) and situated in the *GRIN2D* gene, which encodes a class of ionotropic glutamate receptor and has been associated with schizophrenia susceptibility in the Japanese population (Makino et al., 2005), was not. The two pathogenic-predicted, highly conserved SNVs were located within *LAPTM5* and *SORT1* genes (Table 1; Figure 1(b) and (c))*.* The *LAPTM5* gene encodes for a lysosomal-associated multispanning membrane protein that is known to be associated with spontaneous regression of neuroblastomas, pigmented villonodular synovitis, and lung cancer (Finis et al., 2006; Cortese et al., 2008; Inoue et al., 2009). The *SORT1* gene encodes for sortilin, a member of a family of cellular vacuolar protein sorting 10 domain receptors that is expressed in neurons of the central nervous system, including cortical, hippocampal, and cerebellar neurons, and peripheral nervous system. *SORT1* is known to regulate both neuronal viability and function through its regulation of both protein transport and signal transduction (Willnow et al., 2008) and is known to bind proNTs to control neuronal survival and cessation (Nykjaer and Willnow, 2012). Sortilin, which has multifunctional roles in intracellular trafficking of polipeptide from the golgy apparatus to secretory and endocytic pathways (Nielsen et al., 2001; Chen et al., 2005), has already been implicated in Alzheimer’s disease (AD) and fronto-temporal lobar degeneration. In AD, sortilin has been shown to represent a major endocytic pathway for the clearance of apoE/Aβ complexes (Carlo et al., 2013) and to control APP processing to Aβ (Gustafsen et al., 2013), while in fronto-temporal lobar degeneration it has been shown to regulate extracellular levels of progranulin (PGRN; Hu et al., 2010). Sortilin has also been postulated as a risk factor for hypercholesterolemia and myocardial infarction (Musunuru et al., 2010). Interestingly, all of our *SORT1* mutation carriers were shown later to carry elevated levels of LDL cholesterol, suggesting a possible role of cholesterol homeostasis in the pathogenesis of tremor.

**Table 1.** Heterozygous Disease-Segregating Mutations Identified Through WES. All Variants Were Absent in Large Number of Control Individuals.

Ch	Position	Gene	Nucleotide change	Protein change	Protein domain	Conservation^a^	Pathogenicity prediction^b^	ExAC data (EU fqcy)	Human brain expression^c^	Associated mechanisms/diseases
1	31,211,818	LAPTM5	c.479C > T	p.T160I	Mtp (28-261)	High	Deleterious	Not present	Medium	Lysosomal destabilization/ neuroblastomas, PVNS, autoimmune diseases, and lung cancer
1	109,898,020	SORT1	c.512G > C	p.G171A	Vps10p (133-745aa)	High	Deleterious	Not present	High	NPs ligand, lysosomal sorting, endocytosis/ FTD, heart diseases
19	48,946,504	GRIN2D	c.3321T > A	p.D1107E	None	Medium	Neutral	2/15,812	Medium	Schizophrenia

*Note.* EU fqcy refers to the frequency of the variants in the European population assessed in the ExAC; Mtp refers to Golgi 4-transmembrane spanning transporter.

WES = whole exome sequencing. VPS10p = vacuolar sorting protein 10 p; NPs = neuropeptides; PVNS = pigmented villonodular synovitis; FTD = fronto-temporal dementia; ExAC = Exome Aggregation Consortium.

a Conservation outcomes across different species were taken from the homologene NCBI database (http://www.ncbi.nlm.nih.gov/homologene).

b Prediction of pathogenicity was determined by the following computational programs (See methods): MutPred, SNPs&Go, Mutation Taster, and SIFT.

c Human Brain Expression data were acquired from the Human protein atlas (http://www.proteinatlas.org/).

The entire coding region of *SORT1* was then examined in 61 Spanish ET cases (13 familial and 48 sporadic), but no pathogenic mutation was identified. Likewise, the exon 4 containing the *SORT1* mutation (p.Gly171Ala) was tested in additional 90 ET cases (28 familial ET and 62 sporadic), but no additional mutation carrier was identified.

### Functional Assays

#### The SORT1 p.G171A mutation downregulates its mRNA and protein expression levels

**Figure 3.**
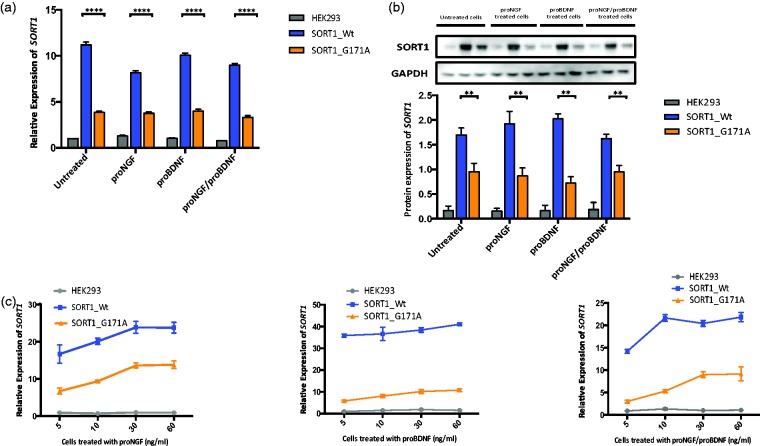
*SORT1* and sortilin expression in untreated and treated cells with proNTs factors. Gray columns represent untrasfected HEK293 cells, blue columns HEK293 cells transfected with *SORT1* wild-type plasmid, and orange columns HEK293 cells transfected with *SORT1* G171A mutant plasmid. Real time PCR analyses for *SORT1* mRNA expression of HEK293 cells untreated and treated with proNTs are shown in (a) (10 ng/ml) and (c) (*x*-axis represents different concentrations of proNGF, proBDNF, and proNGF/proBDNF (5, 10, 30, or 60 ng/ml). The housekeeping gene β2 microglobulin (B2M) was used as control gene. Values represent the mean ± SEM of two different experiments with nine replicates each. (b) Western blot analysis of sortilin and GAPDH proteins in cell lysates obtained from untransfected and transfected HEK293 cells with wild-type and mutant sortilin cultured for 4 days after transfection in the absence and presence of 10 ng/ml of proNGF, proBDNF, and a combination of proNGF/proBDNF. The full-length of sortilin (90 KDa) and GAPDH protein (37 KDa) that was used as a loading control are shown. Results are representative of three separate experiments. Values represent the mean ± SEM. ****p < .0001, while **p < .05.

To further test the pathogenecity of the *SORT1* p.G171A mutation, HEK293 cells were transfected with either wild-type or mutant (p.G171A) plasmids and quantitative PCR (qPCR) on cDNA extracted from wild-type and mutant cells was carried out. Sortilin mRNA levels cells were found nearly three-fold lower in p.G171A mutation carriers than in noncarriers (*p* < .0001; Figure 3(a)), and a similar decrease of two-fold between wild-type and mutant cells was observed at the protein level (*p* < .05; Figure 3(b)). Because sortilin binds to the prodomains of both proNGF and proBDNF to regulate sorting of mature NGF and BDNF to appropriate endocytic and secretory pathways (Chen et al., 2005), transfected cells were also treated with proNGF, proBDNF, and with a combination of proNGF/proBNDF. Sortilin downregulation was consistent in both treated and untreated cells, suggesting that the treatment with proNTs has no effect on its mutant-related differential expression (Figure 3(a)–(c)).

#### The p75^NTR^ mRNA expression is increased in SORT1 p.G171A mutant cells

**Figure 4.**
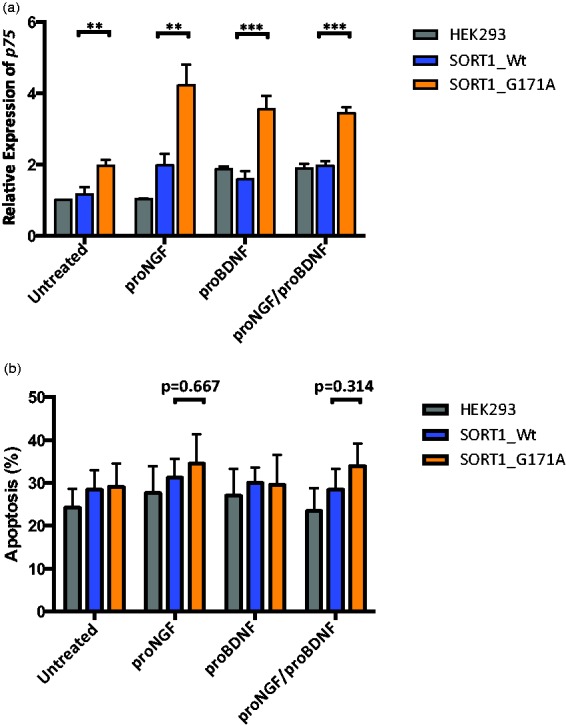
P75^NTR^ mRNA expression and apoptotic cell death levels in wild-type and mutant *SORT1* cells. Gray columns represent untrasfected HEK293 cells, blue columns HEK293 cells transfected with *SORT1* wild-type plasmid, and orange columns HEK293 cells transfected with *SORT1* mutant plasmid. (a) Relative quantification of *p75^NTR^* mRNA expression in untransfected and transfected HEK293 cells with either wild-type or mutant *SORT1* alleles. The *y*-axis shows the relative expression of the target gene (*P75^NTR^*) relative to the internal control gene (*B2M*) and the *x*-axis shows treated and untreated cells with different proNTs (10 ng/ml). (b) Percentage of apoptosis in untransfected and transfected wild-type and mutant *SORT1* cells. The *y*-axis shows the percentage of cells in early apoptosis and the *x*-axis represents different proNT treatments. ***p < .01, **p < .05.

Given the role of sortilin in proNGF- and proBDNF-induced neuronal apoptosis via its interaction with *p75^NTR^* (Nykjaer et al., 2004; Teng et al., 2005) and the effects of the *SORT1* p.G171A mutation on mRNA and protein expression levels, we sought to determine whether this mutation may also alter the *p75^NTR^* mRNA levels and the levels of apoptosis. The expression of *p75^NTR^* in untransfected cells was first tested by direct qPCR: HEK293 cells expressed *p75^NTR^*, and then examined in both wild-type and mutant *SORT1* transfected cells: *p75^NTR^* mRNA levels were significantly increased in mutant cells when compared with their wild-type counterparts (Figure 4(a); *p* < .05, *p* < .01). This increase was more significant in cells treated with proBDNF and proNGF/proBDNF (*p* < .01). Although a slightly increase in apoptotic levels was found in p.G171A mutant cells treated with proNGF and proNGF/proBDNF together, these differences were not statistically significant (Figure 4(b)).

#### PGRN levels are not altered in SORT1 mutant cells or human mutation carriers

**Figure 5.**
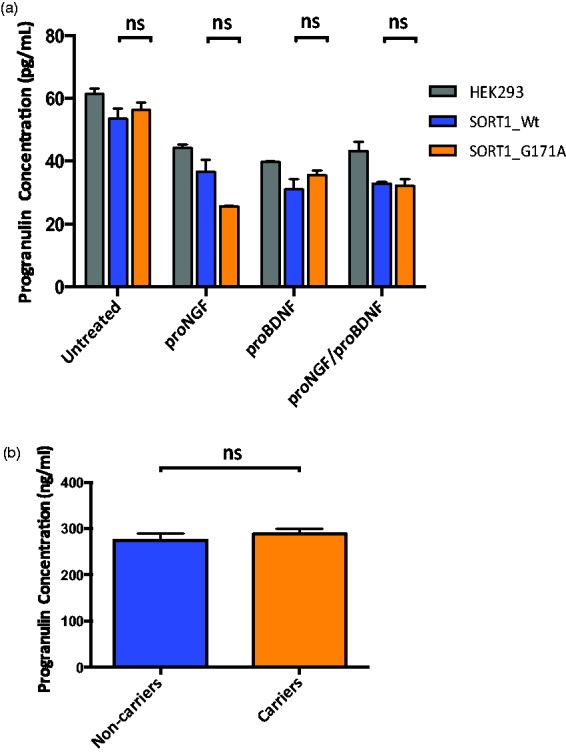
Progranulin expression in HEK293 cells and human serum samples. (a) PGRN levels in cells. Gray columns represent untransfected HEK293 cells, blue columns HEK293 cells transfected with *SORT1* wild-type plasmid, and orange columns HEK293 cells transfected with *SORT1* mutant plasmid. Values on the graph represent the mean ± SEM of three independent experiments. (b) PGRN levels in human serum. The blue column represents PGRN levels for *SORT1* p.G171A noncarriers while the orange column is shown PGRN levels for p.G171A carriers. Values on the graph represent the mean ± SEM of duplicate samples from each individual. Statistical analysis was done with GraphPad software. Non-parametric Mann-Whitney test were performed. *ns* = non significant.

An exciting area of research is the role of sortilin function in the metabolism of PGRN beacuse sortilin has been shown to regulate extracellular levels of PGRN *in vivo* (Hu et al., 2010) and *in vitro* (Lee et al., 2013). Thus, to determine whether the *SORT1* p.G171A mutation, which downregulates *SORT1* mRNA and protein levels and upregulates *p75^NTR^* mRNA levels, may also be associated with increased PGRN levels in culture and human serum samples, PGRN levels were measured by ELISA. No differences in PGRN levels between wild-type and mutant cells (Figure 5(a)) and *SORT1* p.G171A mutation carriers versus noncarriers (*p* = .65; Figure 5(b)) were found.

## Discussion

Despite significant efforts made to identify the genetic basis of ET, the results are limited with linkage, genome wide association, and candidate gene studies failing to detect reliable risk and causative alleles for ET. Here, we examined a family presenting with a mild form of ET characterized by slow progression and beneficial response to alcohol consumption. A disease-segregating mutation resulting in p.Gly171Ala was identified in the *SORT1* gene through WES analyses. Because the expression of sortilin is altered in aging of the nervous system and under pathological conditions (Jansen et al., 2007), we first investigated whether the *SORT1* p.G171A mutation may also alter sortilin expression levels. A highly significant reduction of mRNA and sortilin expression levels was observed in mutant cells when compared with their wild-type counterparts (Figure 3(a) and (b)), suggesting that the *SORT1* p.G171A mutation identified in our ET patients is implicated in sortilin dysregulation. Since the expression of p75^NTR^, which binds to sortilin to mediate neuronal cell death (Nykjaer et al., 2004) and modulates cholinergic transmission (Yang et al., 2002), was also found significantly elevated in mutant cells when compared with wild-type (Figure 4(a)), one may commend that under pathological conditions dysregulation of sortilin may upregulate p75^NTR^ expression levels. Considering that p75^NTR^ is not only upregulated in pathological conditions (Nykjaer et al., 2005) but also in impaired GABAergic transmission (Riffault et al., 2014), the sortilin deficiency caused by the p.G171A mutation might also be responsible for defects in neurotransmission, resulting in both the development of tremor and high p75^NTR^ expression.

Given the role of sortilin as a principal neuronal binding site for PGRN and that a dramatic consequence of such binding is the rapid endocytosis of PGRN by sortilin (Hu et al., 2010), extracellular PGRN levels were also examined. Although PGRN levels were not disrupted in mutant cells versus wild-type (Figure 5(a)) or in mutation carriers versus noncarriers (Figure 5(b)), it has previously been reported that *SORT1* mutants known to disrupt endocytosis do not have altered PGRN levels (Nielsen et al., 2001; Hu et al., 2010), thus leaving the possibility that our *SORT1* mutation could still affect sortilin-mediated endocytosis, sorting, and trafficking, regardless of its effect on PGRN levels.

Lastly, noncoding *SORT1* variation has recently been associated with high cholesterol levels and heart diseases (Musunuru et al., 2010). There are mounting evidence that aberrations in cholesterol homeostasis can lead to severe neurological diseases and impairments in synaptic plasticity (Lane-Donovan et al., 2014); along these lines, high levels of serum total cholesterol have been associated with an increased risk for Parkinson’s disease in Finnish population (Hu et al., 2008). The importance of cholesterol homeostasis for brain function was first highlighted by Niemann-Pick type C disease, in which pathogenic mutations result in impaired cholesterol trafficking and progressive neurodegeneration (Carstea et al., 1997; Naureckiene et al., 2000). The association of a specific apolipoprotein E allele (ɛ4) with the risk for both sporadic and familial AD also raised the possibility that dysfunction in the lipid transport system may cause defects in the brain lipid homeostasis of AD patients (Poirier et al., 2014). Nowadays, it is known that lipoprotein receptors, which participate in synapse development, cargo trafficking, and signal transduction, are key players in AD pathogenesis and neurodegeneration; indeed, the association between impaired lipid metabolism and brain diseases including but not limited to AD, PD and Huntington’s disease is well reported and documented (Lane-Donovan et al., 2014).

In conclusion, we here identified a disease-segregating *SORT1* mutation in a small family with early-onset ET (Figure 1(a)). The pathogenicity of the *SORT1* p.G171A mutation is supported by its absence in neurologically normal population, its prediction as pathogenic by different computational programs, its high conservation across other orthologs (Figure 1(b)), its location in a relevant functional domain (Figure 1(c)), and its effects on both sortilin downregulation (Figure 3(a)–(c)) and p75^NTR^ upregulation (Figure 4(a)). While further research is still required, given the role of sortilin downregulation and p75^NTR^ upregulation in central nervous system impairment and neurodegeneration (Harrington et al., 2004; Jansen et al., 2007; Willnow et al., 2008) and taking into account that changes in the expression level of p75^NTR^ are correlated with functional changes in the GABAergic inhibitory neurotransmission (Blaesse et al., 2009), we hypothesize that the sortilin deficiency caused by the *SORT1* p.G171A mutation may lead to defects in neurotransmission, giving rise to the development of tremor.
